# Efficacy analysis of anterior debridement and bone graft fusion in the treatment of sacroiliac joint tuberculous arthritis: a retrospective analysis of 17 patients

**DOI:** 10.1186/s12891-022-05600-6

**Published:** 2022-07-05

**Authors:** Qi Tian, Cong Peng, Kai Liu, Haopeng Luan, Xiaokang Liu, Linhao Na, Shicong Cao, Zheng Tian

**Affiliations:** Xinjiang Medical University affiliated First Hospital, Ürümqi, China

**Keywords:** Anterior approach, Joint tuberculosis, Sacroiliac joint tuberculosis, Joint fusion

## Abstract

**Background:**

Sacroiliac joint tuberculous arthritis is a relatively rare site of tuberculosis infection, but it can lead to severe sacroiliac joint destruction and dysfunction. Since there are few studies on the surgical methods of sacroiliac joint tuberculosis (SJT), we adopted three different surgical methods based on different degrees of destruction of sacroiliac joint tuberculous arthritis. While revealing its clinical symptoms to improve the diagnostic accuracy, and to determine the safety and feasibility of this surgical approach in the treatment of sacroiliac joint tuberculous arthritis.

**Methods:**

We retrospectively analyzed 17 patients with tuberculous arthritis of the sacroiliac joint treated by anterior debridement. All these patients underwent anterior debridement of tuberculosis with or without bone graft fusion. Mean postoperative follow-up was 17.2 months (12–25 months). The erythrocyte sedimentation rate (ESR) was used to judge the general situation after surgery, and the fusion of sacroiliac joints was observed by X-ray films and CT scans. And VAS and ODI were used to score to observe postoperative functional recovery.

**Results:**

Anterior approach debridement is an effective surgical approach for sacroiliac joint tuberculous arthritis. All patients achieved effective relief of lower back and hip pain. The pain was significantly relieved 3 months after the operation, and the pain basically disappeared 6 months after the operation. The erythrocyte sedimentation rate was also significantly reduced after the operation, and it can basically return to the normal level 3 months after the operation. The VAS score and ODI index of the other 16 patients after surgery were significantly lower than those before surgery, except for 1 patient who died of severe type I respiratory failure and septic shock 3 months after surgery, The surviving patients were basically able to achieve stable fusion of the sacroiliac joint at 12 months postoperatively. None of the patients reported significant pain until the last follow-up visit.

**Conclusions:**

The anterior approach is a very effective surgical method for the treatment of sacroiliac joint tuberculous arthritis, and it is safe and feasible. A clear operative field of view facilitates complete debridement and reduces recurrence, and its function recovers well with stable arthrodesis.

## Introduction

*Mycobacterium tuberculosis* has coexisted with humans for a long time. Before the advent of the new coronavirus pneumonia, *Mycobacterium tuberculosis* was the infectious disease with the highest mortality rate in the world. The Eastern Mediterranean (8.2%), the Americas (2.9%) and Europe (2.5%) have smaller proportions, while the eight underdeveloped countries account for two-thirds of the world’s TB patients, especially Asia, which may Larger, of which China accounts for 8.4% of the world [1], it has become the second most TB country in the world. Musculoskeletal tuberculosis is relatively uncommon, usually bone and joint tuberculosis only accounts for 1–3% of all tuberculosis patients, and about 50% of cases involve the spine, of which only 5–8% involve the sacroiliac joints [2, 3]. Therefore, the low back caused by sacroiliac joint tuberculosis is often overlooked. Sacroiliac joint tuberculosis usually involves the spine or hip joint. Simple sacroiliac joint tuberculosis involvement is very rare. Jatin et al. reported 35 patients with sacroiliac joint tuberculosis [2]. And only 5 to 15% will develop symptoms, the rest have latent infection [3]. Bone and joint tuberculosis is generally characterized by higher local symptoms than systemic symptoms, mainly pain at the lesion site [4]. Sacroiliac joint tuberculosis manifested as pain in the buttocks or lower back. SJT is often misdiagnosed as common lumbar muscle strain and lumbar degeneration and other diseases at the time of diagnosis, because sacroiliac joint tuberculosis is relatively rare and ordinary X-rays are not sensitive to the diagnosis of lesions. Therefore, symptomatic treatments such as anti-inflammatory pain relief and acupuncture and massage were performed, and the doctor did not go to the doctor until there was obvious compression and severe joint pain.

At present, the surgical treatment of sacroiliac joint tuberculous arthritis mainly includes anterior approach, posterior approach, and anterior and posterior approaches combined. The posterior approach is to open a window in the iliac bone to remove the lesion, which is difficult to ensure the thoroughness of the lesion to be removed. At the same time, there is a risk of spreading the tuberculosis foci to the back of the sacroiliac joint. The patient’s health is seriously threatened due to factors such as large trauma, heavy bleeding and long operation time due to the combination of the anterior and posterior approaches. Anterior surgery can fully expose the lesions to completely remove the lesions, with short operation time, less bleeding and fewer complications. Therefore, we introduce 17 patients with sacroiliac joint tuberculosis who underwent anterior debridement to provide some clinical diagnosis ideas and determine the safety and feasibility of surgical treatment.

## Study design and method

### Methods

After receiving the written informed consent from participants and approval from our hospital’s Ethics Committee, We reviewed 17 cases (7 males and 10 females, classified as type III and IV according to Kim’s classification) of sacroiliac joint tuberculous arthritis (SJT) who were treated in the First Affiliated Hospital of Xinjiang Medical University and underwent anterior debridement from June 2012 to June 2021.

Tuberculous sacroiliitis is divided into 4 types by Kim according to X-ray findings and clinical symptoms [5]. Type I only shows widening of the sacroiliac joint space and blurring of the edges; Type II has an eroded articular surface of the sacroiliac joint; Type III presents with severely damaged articular surfaces. Cystic changes or marginal sclerotic changes in the ilium or sacrum; Type IV is characterized by the formation of sacroiliac joint abscesses and even affects other vertebral bodies.

The age of the patients ranged from 13 to 69 years old, with an average age of 25.6 years; 2 cases of type III and 15 cases of type IV. All patients had unilateral infection, 7 cases were on the left side and 10 cases were on the right side; 15 cases were accompanied by abscesses around the sacroiliac joint, and 2 cases were not accompanied by abscesses; Among the infections in other parts, 1 case was associated with pulmonary tuberculosis, 1 case was renal tuberculosis, 1 case was urinary tract tuberculosis and tuberculous meningitis, and 14 cases were not associated with tuberculosis in other parts. The average time from the onset of symptoms to seeing a doctor was 18.6 weeks, the shortest was 2 weeks, and the longest was 14 months (see Table 1). Each patient signed a consent form for data collection. And this study was approved by the First Affiliated Hospital of Xinjiang Medical University.

**Table 1:** Patient’s basic information

value	Gender	Age	Infected Side	other infected sites	abscessus	Kim’s Type	Treatment of surgery
1	F	28	L	No	N	III	A
2	M	25	R	No	Y	IV	A
3	M	25	L	No	Y	IV	C
4	F	17	L	No	Y	IV	B
5	F	21	R	No	Y	IV	A
6	F	20	R	No	Y	IV	C
7	F	16	L	No	Y	IV	A
8	M	19	L	No	Y	IV	C
9	M	15	R	No	Y	IV	C
10	F	32	L	No	Y	IV	A
11	F	13	R	No	Y	IV	C
12	F	26	R	Kidney	Y	IV	A
13	M	23	R	No	Y	IV	B
14	M	16	L	No	N	III	A
15	F	36	R	No	Y	IV	B
16	F	69	R	Urinary system	Y	IV	C
17	M	34	R	No	Y	IV	B

Pain in the lower back, buttocks and hip joints leading to difficulty walking was the most common symptom in our study. Thirteen of them were accompanied by difficulty walking and could not walk with full weight.7 cases of pain were mainly in the proximal thigh and buttocks, One case presented with hip pain radiating to the heel. Other non-specific manifestations of tuberculosis included 7 cases of low fever in the afternoon, 8 cases of night sweats, 4 cases of decreased appetite, and 5 cases of recent significant weight loss. Only 1 patient had a palpable mass on the body surface; physical examination found that all patients had obvious tenderness of the affected side of the sacroiliac joint. Patrick’s test, Gaenslen’s test, and Yeoman’s test on the affected side were all positive. Significant limitation of movement of the affected limb, with hyperextension and flexion of the affected sacroiliac joint, the movement of the affected joint is markedly limited and severe pain is caused during movement. After the patient is admitted to the hospital, a preliminary diagnosis of sacroiliac joint tuberculous arthritis is made based on typical clinical symptoms combined with blood routine, erythrocyte sedimentation rate, PPD test, test, T-SPOT, TB-DOT test and anti-tuberculosis antibody tests. At the same time, combined with pelvic anteroposterior X-ray, sacroiliac joint CT and MRI scan for further verification, However, the final diagnosis needs to be confirmed by mycobacterial culture or pathological examination of fine needle aspiration or intraoperative curettage. In highly suspected sacroiliac joint tuberculosis infection or confirmed by fine needle aspiration biopsy of sacroiliac joint tuberculosis infection, quadruple anti-tuberculosis drug treatment is usually required immediately. Oral isoniazid once/d, total 300 mg/d, rifampicin once/d, total 450-600 mg/d, pyrazinamide 1–3 times/d, total 1500-1750 mg/d, ethambutol 1–2 times/d, a total of 750-1000 mg/d. Active nutritional support therapy should be given while controlling body temperature, ESR and other symptoms. Preoperative drug anti-tuberculosis treatment for about 2 weeks. Surgery to remove the lesion when the erythrocyte sedimentation rate is below 40 mm/h. Even if the erythrocyte sedimentation rate is still higher than 40 mm/h, patients with abscess or other vertebral tuberculosis can be treated with surgery after excluding active pulmonary tuberculosis.

**Fig. 1:**
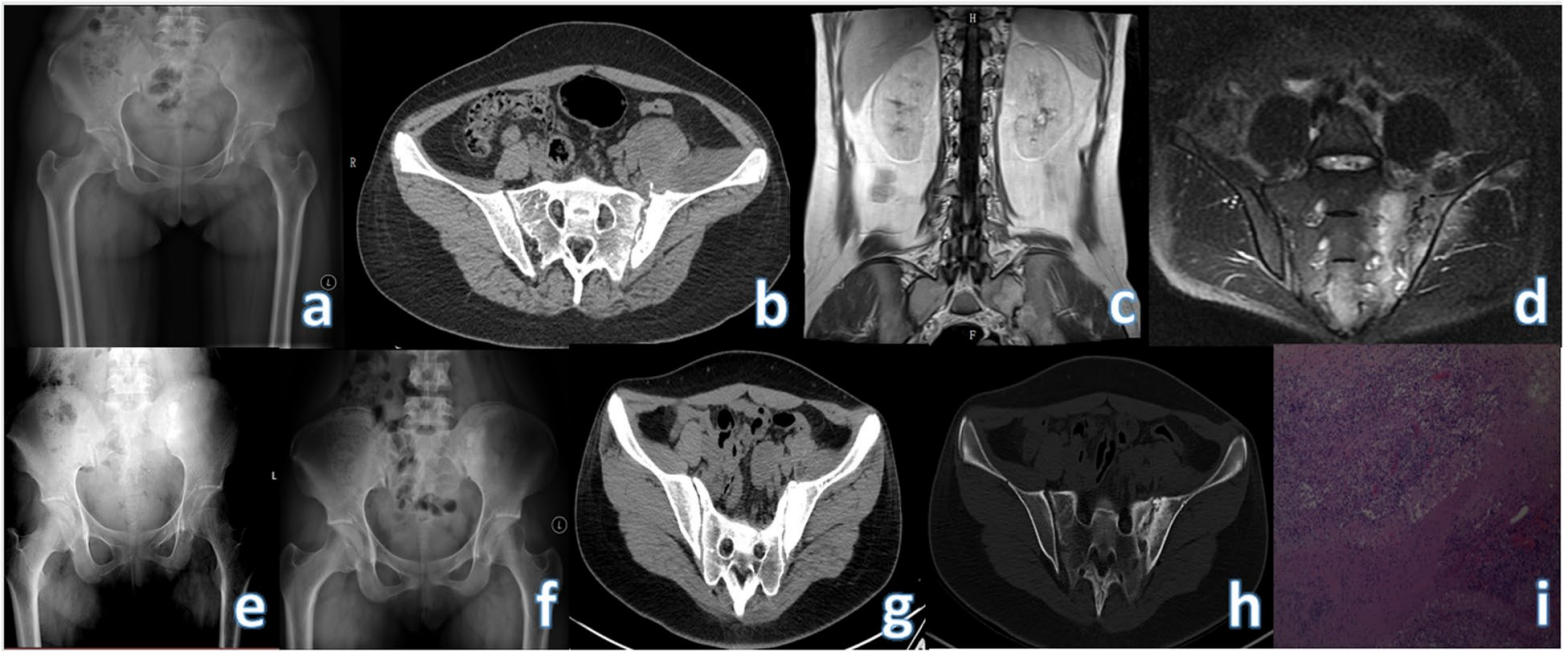
Preoperative X-ray, CT and MRI of the patient, as well as X-ray at 3 months, 6 months after operation and CT at 12 months after operation of NO.4. **a-d** Preoperative X-rays, CT and MRI: the right sacroiliac joint space is indistinct and part of the bone is destroyed; the left iliac muscle and the front of the hip joint can be seen with irregular and mixed density soft tissue shadows; the psoas major muscle can be seen with patchy slightly Long T2 signal. **e** X-ray at 3 months after operation: it can be seen that the bone graft is not fused with the joint. **f** X-ray at 6 months after operation shows the fusion of the bone graft and the joint. **g-h** There is no bone resorption in the bone graft part, and the joint part is fused in the CT at 12 months after operation

**Fig. 2:**
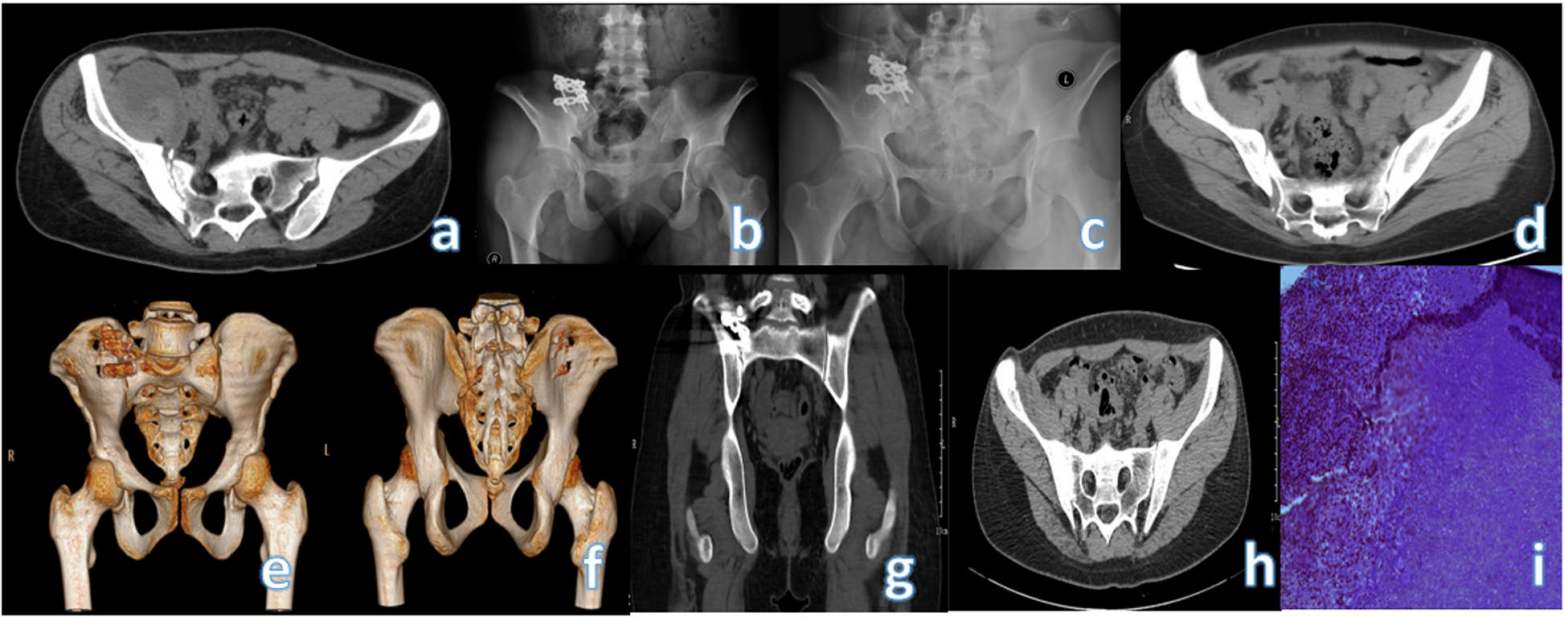
Preoperative CT, X-ray at 3 months after operation, X-ray at 6 months after operation, and CT at 12 months after operation of NO.6. **a **The right sacroiliac joint space is widened, the articular surface is not smooth and tidy, the band-like bone density is increased, and the articular surface of the iliac flank is destroyed by insects. **b** The implanted autologous ilium did not see obvious bone resorption occurred 3 months after surgery. **c** Part of the bone in the operation area can be seen fused 6 months after the operation, and the bone density in the operation area is not uniform. **d**-**h** Three-dimensional reconstruction imaging of the sacroiliac joint showed that a relatively stable arthrodesis had been achieved 12 months postoperatively

Based on preoperative imaging findings, we performed anterior tuberculous debridement with or without bone graft fusion in 17 patients with sacroiliac joint tuberculous arthritis. There are three types of surgical methods: surgical approach of type A is simple debridement. It is suitable for those with no obvious widening of joint space and small defect after curettage; The surgical approach of type B is the use of autologous iliac bone grafts to fuse the joint after curettage, (Fig. 1). It is suitable for those with larger articular surface defects after curettage;Type C method is to use Titanium locking steel plate and screws to fix on the basis of B method (Fig. 2), It is suitable for unstable or even dislocation of the sacroiliac joint after surgery. For patients with unstable sacroiliac joints after lesion removal, internal fixation devices should be used to enhance the stability of the joints. Among them, 6 cases used plate internal fixation to strengthen the sacroiliac joint.

The anterior approach was used in all operations, and the patients were kept in the supine position or the lateral position with elevated hips. An arc-shaped incision of about 9 cm in length was made from the anterior superior iliac spine to the highest point of the iliac wing and along the iliac crest as the surgical approach. If there is a sinus tract, expand and cut along the sinus tract to the abscess. If there is no sinus tract, determine the location based on the location of the abscess. Pay attention to protect the lateral femoral cutaneous nerve, ureter, and deep iliac vessels. The contents of the abdomen and pelvis were retracted inward, and gauze was used to protect the surrounding tissue of the abscess. The pus should be carefully drawn out by using a syringe. Re-blunt dissection of the abscess for further complete aspiration. Then expand the abscess incision and continue to explore deep. Incision of the iliac crest and iliacus muscle and continued separation to the front and upper part of the sacroiliac joint. Curettage of sacroiliac joints of tuberculosis, inflammatory granulomas, and complete removal of sequestrum until normal bone appears. And use carbolic acid and absolute ethanol to inactivate local lesions. Send the aspirated material and scraped material to histopathological examination in time, and use hydrogen peroxide and normal saline to rinse the operation area repeatedly. Sprinkle 2 g of streptomycin on the scraped area. Autologous iliac bone or allogeneic artificial bone is implanted into the joint space. If the stability is still poor, the stability of the joint is reconstructed with a plate and screw internal fixation system. Drainage tubes are to be placed in the surgical area. The drainage tube was not removed until 3 days after surgery.

Continue to use the same quadruple antituberculosis therapy after surgery for a total of 18 to 24 months, Liver and kidney function needs to be checked regularly during the postoperative period, and the toxic effects caused by the side effects of anti-tuberculosis drugs should be found at any time. The indicators monitored during the follow-up period included liver and kidney function, erythrocyte sedimentation rate, joint fusion of X-ray and CT scan, VAS and ODI index. The VAS and ODI data before operation, 6 months after operation, and 12 months after operation were analyzed by SPSS software, and Wilcoxon rank sum test and Mann-Whitney test were used. *P* ≤ 0.05 was considered to be statistically significant.

## Results

On imaging, CT showed narrowing of the joint space and erosion of the articular surfaces. In patients with abscesses, soft tissue shadows with irregular shapes and mixed density were seen near the sacroiliac joints and surrounding musculature. MRI is more accurate, showing a patchy slightly longer T1 signal shadow of the sacroiliac joint bone, showing high signal on the fat-suppression sequence, and a patchy slightly longer T2 signal in the abscess near the muscle tissue. Pelvic fluid signs. During the operation, caseous necrotic tissue and sequestrum were found in all cases, which caused severe invasion and damage to the articular surface, and the intraoperative exploration was consistent with the preoperative MRI. The location of the abscess is consistent with the results of the preoperative examination, and it is located in the anteromedial or anterolateral sacroiliac joint. One of the patients (case NO.3) was seen again 3 months after the operation due to the formation of the inguinal sinus, and we expanded and removed the sinus again along the sinus. Since the sacroiliac joint was found to be stable, we removed the internal fixation plate while removing the abscess. Complete sacroiliac fusion at 12 months postoperatively. All patients were finally confirmed as sacroiliac joint tuberculous arthritis by pathological examination or bacterial culture of *Mycobacterium tuberculosis*.

The mean operation time of all patients was 105.6 ± 39.9 min (60-200 min), and the mean estimated blood loss was 258.8 ml (50--1500 ml) (Table 2). Except for 1 patient who died of type I respiratory failure and septic shock 3 months after the operation due to urinary tract tuberculosis, tuberculous meningitis and severe pulmonary infection. The symptoms of the remaining 16 patients were significantly improved after surgery. The average preoperative ESR was 58.6 ± 13.7 mm/h (32-80 mm/h), which decreased to 38.7 ± 8.8 mm/h (26--50 mm/h) within 7 days after surgery.), erythrocyte sedimentation rate was 10.4 ± 6.1 mm/h (5-24 mm/h) 3 months after operation. Although patient No.6 had an ESR of 24 mm/h at 12 months after surgery, she did not express any discomfort; We believe that patients with only high ESR after surgery should continue oral antituberculosis drugs. If there is not only high ESR but also other symptoms such as local pain after surgery, recurrence and timely surgical intervention should be considered. Through the X-ray or CT of the sacroiliac joint after the operation, it was observed that the patient could be relieved at about 3 months after the operation. Signs of bony fusion of the sacroiliac joint were observed (Fig. 1).CT was more obvious than X-ray, and further fusion was observed at 6 months postoperatively (Fig. 1). All patients were able to walk with full weight bearing without the aid of crutches at the last follow-up visit. They were all able to regain their flexion and extension ability, walked 300 m on flat ground or up and down 2-story stairs without crutches, and showed no significant discomfort such as pain. The VAS scores of all 16 patients were significantly decreased after operation (Table 3), and there were significant differences between preoperative and postoperative 3 months, 6 months and 12 months after operation (*P* < 0.05). Until the last follow-up, only 1 patient (No. 3) visited the doctor due to sinus tract in the groin and underwent secondary surgery, and the remaining patients did not have recurrence.

**Table 2:** Results of 17 patients

outcome	Surgical management (*n* = 17)		
Intraoperative blood loss (ml)	361.8 ± 630.2		
Intraoperative blood transfusion (case)	4		
operation time (min)	105.6 ± 39.8		
ESR	Preoperative	7 days after surgery	3 months after surgery
	58.6 ± 13.7	38.7 ± 8.8	10.4 ± 6.1

The normal reference value of ESR in our hospital: < 15 mm/h (male), < 20 mm/h (female)

**Table 3:** VAS and ODI results of 16 patients

outcome	VAS and ODI(*n* = 16)			
VAS	Preoperative	3 months after surgery	6 months after surgery	12 months after surgery
	6.6 ± 1.5	3.2 ± 0.6	2.1 ± 0.6	1.3 ± 0.4
ODI	56.1 ± 4.2	39 ± 4.8	27.6 ± 5.7	4.3 ± 1.6

## Discussion

The sacroiliac joint is a joint complex composed of the surrounding soft tissues such as the sacrum, the auricular surface of the ilium, cartilage and ligaments. The soft tissue between the sacroiliac joints is divided into the anterior 1/3 of the synovial tissue and the posterior 2/3 of the ligament structure. Since the anterior 1/3 of the synovium is composed of the complete joint capsule, articular cartilage and joint space, the sacroiliac joint is a true joint. When *Mycobacterium tuberculosis* enters the blood, it enters the cancellous bone and joint synovium with less muscle attachment and rich blood vessels through the circulatory system, causing the tuberculosis synovial flora and abscess of the sacroiliac joint to further erode the articular cartilage and destroy the ilium and sacroiliac joint. Its unique anatomical characteristics make sacroiliac joint tuberculosis mainly in the synovial part of the sacroiliac joint. Compared with the sacral side, the hyaline cartilage on the surface of the iliac bone is thinner [6]. And the lesions involve the synovium of the anterior and lower parts of the sacroiliac joint, and the lesions on the iliac side are more serious than those on the sacral side.

Since the sacroiliac joint complex is part of the posterior ring of the pelvis, the posterior ring structure contributes 60% to the stability of the pelvis, and is an important structure for transmitting the load of the trunk and lower limbs and completing the mechanical conduction. The current study suggests that the sacroiliac joint has limited flexion and extension movements within an average range of 3°, followed by axial rotation (about 1.5°) and lateral bending (about 0.8°).The sacroiliac joint is a movable joint from an anatomical point of view, but it is a micro-movement joint sacroiliac joint when viewed functionally [7]. Therefore, the sacroiliac joint is very important for the overall stability of the human body. The pain distribution of sacroiliac joint tuberculosis is not fixed because the abscess can move in any direction and compress or stretch the lumbosacral nerve. And the early lesions are mainly in fibrocartilage and synovium, so the early symptoms of sacroiliac joint tuberculosis are not obvious. Most patients present with complaints of lower back pain and difficulty walking. And only a few people have typical tuberculosis symptoms such as low fever in the afternoon, night sweats, recent significant weight loss and poor appetite. In our study, we found 7 cases of low fever in the afternoon, 8 cases of night sweats, 4 cases of decreased appetite, and 5 cases of recent significant weight loss. Therefore, it is often misdiagnosed as lumbar muscle strain and lumbar disc herniation in the early stage, so that the disease is delayed due to symptomatic physical therapy such as acupuncture, massage and oral painkillers. In the end, the operation was performed because of obvious pain and difficulty in walking. The course of sacroiliac joint tuberculosis is slow. In the late stage, serious complications such as instability of the sacroiliac joint and upward dislocation of the pelvic ring may occur.

In the early X-ray examination of sacroiliac joint tuberculosis, it is necessary to compare bilateral sacroiliac joints, and raise the affected buttocks by about 15° to fully display the joint surface. Blurred articular surfaces and widening of the joint space may be observed. As the disease progresses, the joint space gradually narrows or even disappears, sequestrum forms, accompanied by increased articular surface bone density and even cystic degeneration. Early CT can show thickening of the synovium and destruction of the smaller round or wedge-shaped bone around the joint. Widening and blurring of the sacroiliac joint can be found when compared to the uninjured side. Invasion of surrounding soft tissue and muscle can be shown more clearly than X-ray. This greatly improves the early diagnosis rate. Bone edema and bone destruction can be seen early in the disease on MRI. It is an ideal examination to observe soft tissue lesions around the joints and the appearance of abscesses. However, the final diagnosis depends on histopathological examination or *Mycobacterium tuberculosis* culture. Kim divides the sacroiliac joint tuberculosis injury into four types according to the severity of the injury. Types I and II are relatively mild, and it is generally recommended to give anti-tuberculosis drugs for symptomatic treatment first. For type III and IV with severe joint destruction, cystic degeneration of ilium or sacrum, marginal sclerosis and even formation of sacroiliac joint abscess, surgical treatment is required. Because this type of lesion is prone to spread to other locations and instability of the sacroiliac joint, adequate drainage of pus and removal of the lesion with bone graft fusion is beneficial to achieve joint stability at an early stage. The surgical indications for debridement of sacroiliac joint tuberculosis are generally sacroiliac joint tuberculosis with large tuberculosis abscess or sequestrum formation. Surgical methods generally include anterior debridement, posterior fenestration debridement, and combined anterior and posterior approaches. Anterior approach is suitable for abscesses or sequestrum mainly anterior to the sacroiliac joint. Incomplete debridement of anterior iliac abscess by posterior approach, and there is also a huge risk of spreading the TB lesions from the front to the back. According to the results of preoperative imaging examinations and the preoperative planning of surgery, all 17 patients underwent debridement through the anterior approach. Anterior debridement surgery has the following advantages. (1) Short operation time, less bleeding, and less trauma (2) The entire operation is performed under direct vision, which is easy to clearly identify the anatomical structure and reduces the possibility of injury to important blood vessels, nerves and ureters in the pelvis. ③ The anterior sacroiliac joint, the anterior lumbosacral and bilateral tuberculosis abscesses can be fully exposed and completely removed. ④ The stability of the sacroiliac joint is obviously protected, because only the anterior ligament is damaged by the lesion, and the unbroken posterior ligament is preserved. ⑤ Compared with posterior fenestration surgery, it can remove tuberculosis lesions more thoroughly and prevent the possibility of recurrence to the greatest extent. ⑥ Anterior plate fixation requires less fluoroscopy than posterior fixation, which is a kind of protection for both the operator and the patient.

Therefore, anterior approach is easier to remove sacroiliac joint lesions than posterior iliac fenestration. However, the operating space of anterior surgery is narrow. Tribus and colleagues conducted an anatomical study of 35 cadaveric specimens and showed that the average distance from the bifurcation of the iliac vessels to the L5S1 intervertebral space was only 1.8 cm. The average transverse distance of the common iliac artery is 3.4 cm, and the surgical operation space is small [8]. so the requirements for the operator are relatively high. Therefore, the familiarity of the surgical approach should be fully assessed before surgery, the risk of surgery should be assessed, the empty needle should be withdrawn from the abscess, and the scope of exposure should be expanded when there is only pus but no blood returning. Blunt dissection is a relatively safe method.

Because tuberculosis infection is a physical wasting disease, sacroiliac tuberculosis patients generally have poor physical fitness and lower hemoglobin levels. During the operation, attention should be paid to monitoring the hemoglobin level, and if necessary, blood transfusions should be given in time to avoid accidents.

The difficulty of the anterior approach is that it is accompanied by important anatomical structures such as the common iliac artery and vein, the ilioplumbar artery and vein, the middle sacral artery and vein, and the urinary and reproductive systems. Due to the occlusion of these important structures, it is difficult to fix the implant. Finally, postoperative complications such as abdominal distension, constipation, and ejaculation disturbance may occur. However, in general, anterior approach surgery is the best surgical approach for patients with sacroiliac joint tuberculosis and anterior abscess, and the combined approach is still an indispensable and effective supplement. It is Anterior open surgery that is indeed a morbid surgery compared to minimally invasive surgery [9, 10]. However, we still believe that an anterior open surgery is necessary to allow adequate curettage with adequate visualization. Murakami et al. exposed the superior anterior surface of the sacroiliac joint by separating between the psoas major and iliacus muscles [11]. However, we believe that by wrapping the dissected iliac muscle around the femoral nerve and surrounding neurovascular, and pulling them together to the opposite side, it is more beneficial to protect them.

Ahmed et al. believe that in acute cases, the main purpose should be to salvage the integrity of the joint through early debridement according to joint destruction and general patient conditions; when it is chronic, debridement of the joint alone is not safe and should be fused [12]. Yu et al. believed that the sacroiliac joint has a large area of damage (> 50%) or is accompanied by instability, deformation or dislocation of the sacroiliac canal joint. On the basis of bone graft fusion, internal fixation with a reconstructed plate is required to stabilize the sacroiliac joint [13]. We believe that a large cavity will be left after the lesion is removed, which often causes joint instability and chronic pain after surgery. Since the sacroiliac joint is a micromotion joint, The range of motion in flexion and extension is approximately 3°, and the ranges of axial rotation and lateral flexion are 1.5° and 0.8°respectively [7]. In our study, Instability of the sacroiliac joint is defined as an angle greater than these ranges during passive movement during surgery. Patients with no obvious widening of the joint space and small defect after curettage were treated with operation A, those with larger articular surface defects after curettage were treated with operation B, and those with unstable or even dislocated sacroiliac joints were treated with operation C. treatment.

Bone grafting can promote joint fusion and further enhance stability. Except for 1 patient who died, the remaining 5 patients underwent plate removal after surgery, and they did not feel pain and other discomfort at the last follow-up. In our study, we found that postoperative VAS and ODI were significantly lower than preoperative (Table 3). All patients were able to move without bracing at 3 months postoperatively and some light exercise at 6 months.

In general, the anterior approach can completely remove the infection lesions through a clear field of vision, but it cannot completely replace the treatment of anti-tuberculosis drugs. After surgery, you should continue to take quadruple anti-tuberculosis drugs. The total course of treatment should be 18 to 24 months. Therefore, anterior debridement is an efficient and safe surgical method for the sacroiliac joint, but a large number of clinical trials and multicenter trials are still needed for further verification.

## Data Availability

The data sets generated and analyzed during the current study are not publicly available due to restrictions on ethical approvals involving patient data and anonymity but can be obtained from the appropriate authors as reasonably required.
